# Civil Monetary Penalties from Violations of the Emergency Medical Treatment and Labor Act for Patients Arriving or Leaving with Law Enforcement

**DOI:** 10.5811/westjem.39677

**Published:** 2025-05-19

**Authors:** Sameer Ahmed, Zach Reichert, Genevieve Santillanes, Carmen Toomer, Sandra Tyler-Mills, Neha Vontela, Jasmine Hsia, Sarah Axeen, Saman Kashani, Joe Nakagawa, Michael Menchine, Sophie Terp

**Affiliations:** * Keck School of Medicine of the University of Southern California, Department of Emergency Medicine, Los Angeles, California; † Los Angeles General Medical Center, Department of Emergency Medicine, Los Angeles, California; ‡ University of Southern California, Leonard D. Schaeffer Center for Health Policy and Economics, Los Angeles, California; § Los Angeles County Fire Department, Los Angeles, California; || City of Hawthorne Police Department, Hawthorne, California

## Abstract

**Introduction:**

The Emergency Medical Treatment and Labor Act (EMTALA), a federal law enacted in 1986, is intended to prevent inadequate, delayed, or denied treatment of emergency medical or emergency psychiatric conditions by Medicare-participating hospitals when individuals present to dedicated emergency departments (EDs). EMTALA requires all patients seeking evaluation for an emergency medical condition (EMC) at a dedicated ED to have an appropriate medical screening exam (MSE), stabilization of identified EMCs, and an appropriate transfer if specialized services are needed for stabilization.

**Methods:**

We obtained summaries of all EMTALA-related civil monetary penalties (CMPs) between 2002–2023 from the Office of the Inspector General (OIG) and reviewed them for instances where patients arrived or departed with law enforcement officers (LEOs). In this article, we describe the characteristics of these CMPs.

**Results:**

Of 260 EMTALA-related CMPs, 15 (5.8%) were identified as having involved patients arriving to or departing from an ED with LEOs. Among these, nine (60%) involved patients arriving to the ED with LEOs, of whom five (55.6%) were transported to alternate facilities by LEOs at the direction of ED staff without receipt of an appropriate MSE. Overall, eight (88.9%) of nine patients arriving with LEOs involved psychiatric concerns. Four cases were identified as having involved patients discharged from but not arriving to the ED with LEOs. Of these, two involved patients brought to the ED for evaluation of psychiatric conditions and discharged to jail without appropriate MSE after becoming disruptive. Two involved patients with psychiatric issues sent to jail without appropriate MSE/stabilization, some due to hospital policies pertaining to alcohol intoxication. Two involved patients without noted psychiatric concerns escorted from the ED with the assistance of LEOs after reported to be “resistant” or “aggressive.” One returned to the ED in cardiac arrest, and another was subsequently diagnosed with bacterial meningitis.

**Conclusion:**

Overall, 5.8% of EMTALA-related CMPs involved patients arriving to or departing from the ED with LEOs; most of these involved patients with psychiatric emergencies. In many cases, LEOs were advised to either transport patients to an alternate medical facility without an appropriate MSE, or disruptive or intoxicated patients with noted psychiatric concerns were discharged to jail without adequate MSE or stabilization. Findings indicate a need for education surrounding EMTALA requirements to provide MSEs and, if needed, stabilizing treatment prior to discharge or transfer for all patients presenting to the ED, regardless of LEO involvement.

## INTRODUCTION

Interactions between patients and law enforcement officers (LEOs) occur frequently in emergency departments (EDs).^1^ Individuals in the custody of LEOs requiring emergency medical care are commonly brought to EDs similarly to individuals requiring medical clearance for booking at a correctional facility. LEOs often respond to psychiatric and mental health emergencies in the prehospital setting and transport patients to EDs for evaluation of behavioral or psychiatric emergencies.^2^–^4^ Further, LEOs are also sometimes called by the ED to ensure the safety of staff or other patients when patients are perceived to be aggressive or violent and may be used to assist in removal of patients who are violent, threatening, or refuse to leave the hospital premises.^3^–^6^ Despite frequent interactions between LEOs and hospital staff, each may approach encounters with ED patients with different perspectives and priorities.

Many of a hospital’s duties pertaining to the care of patients requiring evaluation or stabilization of emergency conditions are specified in the Emergency Medical Treatment and Labor Act (EMTALA), which is intended to prevent inadequate, delayed, or denied treatment of emergency medical or emergency psychiatric conditions.^7^ Regardless of LEO presence or requests, EMTALA requires all hospitals with a Medicare agreement to provide patients for whom evaluation or treatment for an emergency condition is sought with the following: 1) an appropriate medical screening exam (MSE), 2) stabilization of any identified emergency medical condition (EMC), and 3) appropriate transfer to another facility if services required for stabilization are not available at the initial hospital.^7^ EMTALA is actively enforced, with nearly half of hospitals in the United States investigated and two-thirds of investigated facilities cited by the Centers for Medicare & Medicaid Services (CMS) for a violation between 2005–2014.^8^ A summary of the EMTALA enforcement process is included in Supplementary Exhibit A. Additionally, the Department of Health and Human Services Office of the Inspector General (OIG) can impose civil monetary penalties (CMP) to facilities found to have negligently violated EMTALA.^9^ Prior published manuscripts have described general as well as hospital-level characteristics associated with EMTALA-related CMPs, as well as EMTALA-related CMPs involving individual physician fines, pediatric emergencies, psychiatric emergencies, and obstetric emergencies.^10^–^15^

While reviewing CMP summaries for prior related studies, we incidentally noted that they often described scenarios involving individuals arriving to or departing from the ED accompanied by LEOs.^12^–^15^ Hospitals have the same EMTALA obligations to an individual in law enforcement custody as any other individual who comes to the ED. Similarly, an individual being placed under custodial arrest while receiving care in an ED does not terminate EMTALA obligations. EMTALA obligations are the responsibility of the hospital, not law enforcement. An understanding of prior CMPs may serve to inform hospitals in their efforts to ensure EMTALA compliance when serving this particularly vulnerable population. The purpose of this study was to inform clinicians and hospital administrators working at Medicare-participating hospitals how EMTALA has been enforced by describing cases where involved patients were noted to arrive or depart from the ED with LEOs.

**Population Health Research Capsule**

What do we already know about this issue?

*Patients in the ED often interact with law enforcement officers (LEOs). EMTALA established a duty for hospitals to screen and stabilize patients presenting to an ED.*

What was the research question?

*Our goal was to describe EMTALA-related civil monetary penalties involving LEO-involved ED patients.*

What was the major finding of the study?

*Fifteen of 260 cases involved LEO-involved patients, 12 with psychiatric concerns. Four were discharged to jail without a medical screening exam/stabilization.*

How does this improve population health?

*Emergency clinicians may benefit from education regarding EMTALA requirements to evaluate, stabilize and, when necessary, transfer LEO-involved ED patients.*

## METHODS

We obtained summaries of all EMTALA-related CMPs occurring between 2002–2023 from the OIG website.^16^ Consistent with methodology applied in prior studies using this data, CMPs related to EMTALA violations were identified by the inclusion of “EMTALA” or “patient dumping” in the summary text.^12^,^14^,^15^ Semiannual reports published by the OIG were additionally reviewed to ensure accuracy and completeness of the dataset.^16^,^17^

We systematically reviewed all 260 CMP settlement summaries for instances where patients were specifically noted to have arrived to or departed from the ED accompanied by law enforcement personnel (which we will refer to as LEO-involved) using keywords and stems: “law enforcement, police, sheriff, deputy, jail, prison, criminal-, detain-, charge-.” Of note, summaries varied in the level of granularity of case details provided, and as a result some cases involving LEOs may not have been identified. Summaries were reviewed for context indicating that an involved patient was in the custody of law enforcement at the time of the ED visit or otherwise had arrived to or departed from the ED with law enforcement personnel.

Summaries of all 260 CMPs that occurred within the study period were reviewed by three trained research assistants and flagged if reference of LEO-involvement was identified. Of the 260 CMPs, 17 CMPs were flagged and subsequently reviewed by a team of three practicing emergency physicians who determined that 15 met criteria for inclusion within the study (included in Supplementary Exhibit B), one additionally described the threat of LEO-involvement (included in Supplementary Exhibit C), and a final CMP only referenced a patient having been found down by LEOs in the field but who was not specifically noted to have arrived to the ED with LEOs or to have been in custody of LEOs. The CMP summaries describing LEO-involved patients were reviewed for the following key characteristics: 1) whether a patient was noted to arrive to the ED with a LEO, 2) patient features including age or condition, 3) whether a patient departed from the ED with a LEO, and 4) types of EMTALA deficiencies noted in the narrative CMP summary.

This study evaluating CMPs using publicly available data was determined not to be human subjects research by the Institutional Review Board of the University of Southern California.

### Illustrative Case Studies

Inspection text from the EMTALA citation events preceding two of the CMPs involving patients arriving to or departing from the ED with LEOs were obtained from publicly available CMS hospital inspection data. These cases were selected by author consensus to highlight key learning points. For each, we summarized details of the EMTALA violation, investigation findings, and CMP findings to provide a richer understanding of the EMTALA enforcement and CMP settlement process.

## RESULTS

Between 2002–2023, there were 260 CMPs related to EMTALA. Among these, 15 (5.8%) summaries described LEO-involved cases. A total of 17 patients are represented among the 15 CMPs; 13 CMPs involved a single patient, and two CMPs involved two patients each (Table 1). Overall, 15 (100%) CMPs referenced failure to provide appropriate MSE, and 10 (66.7%) referenced failure to provide stabilizing treatment. Only two specifically referenced penalties as being related to a failure to arrange appropriate transfers. Illustrative cases are included in Tables 2 and 3.

Nine CMPs (60%) involved patients arriving to the ED with LEOs, including eight patients (88.9%) with psychiatric concerns. Of these nine, five (55.6%) were transported to alternate facilities by LEOs at the direction of ED staff without receiving an appropriate MSE. One ED turned away paramedics transporting an incarcerated patient for whom the hospital had a “no trespass” order. Three CMP summaries described scenarios in which patients arriving to an ED with LEOs were discharged from the ED without LEOs. In one case, a patient brought to the ED by LEOs was placed on an involuntary hold and discharged five hours after ED arrival without the hold being lifted. Another involved a patient brought to the ED with unusual behavior and incoherent speech who was diagnosed with “altered mental status” and “mental health disorder” who was discharged without an appropriate MSE or stabilizing treatment after reportedly yelling and making threatening gestures. A third involved a patient held involuntarily in an ED for 38 days prior to discharge without management by an on-call psychiatrist or admission to an available inpatient bed for stabilizing care.

Four CMPs described patients who did not arrive to the ED with LEOs but were arrested in the ED and discharged to LEO-custody without appropriate MSE and/or stabilization of an EMC. All four involved one or more patients noted to display signs of psychiatric emergency conditions, or to be on an involuntary psychiatric hold. One CMP involved two patients, one of whom had an emergency psychiatric condition and was not provided with stabilizing treatment before being arrested and transferred to jail; a second patient not specified to have a psychiatric condition was not provided with an appropriate MSE before being arrested and sent to jail. Additionally, CMPs were levied against two hospitals after patients awaiting evaluation for psychiatric concerns were arrested after becoming physically aggressive in the ED and discharged without receiving an MSE and/or stabilization of an EMC.

One CMP involved two patients who presented to the ED for intentional overdose, both of whom also had concurrent alcohol intoxication. Both patients were discharged to jail per the hospital’s policy regarding the transfer of patients with blood alcohol concentrations above a specified threshold by LEOs, in this case without MSE/stabilization. One of these patients, who returned to the hospital shortly thereafter following a repeat overdose attempt, required hospitalization in the intensive care unit (ICU).

Two CMPs involved patients without noted psychiatric issues who were escorted out of the ED with the assistance of LEOs. One case involved a patient with a headache who was escorted out by LEOs after acting agitated and resistant when asked to leave the waiting room. Emergency medical services (EMS) was called from the medical center parking lot, where the patient was found unresponsive, and they transported her to another facility where she was diagnosed with bacterial meningitis, requiring mechanical ventilation and hospitalization in the ICU. Another CMP involved a patient who was escorted off the property by LEOs after exhibiting “aggressive behaviors” shortly after arriving to the ED by ambulance. The patient returned to the ED five hours later in cardiac arrest and ultimately died. Additionally, a case in which hospital staff threatened to involve LEOs but did not do so is not included in analysis but is described in Supplementary Exhibit C.

## DISCUSSION

Between 2002–2023, 5.8% of EMTALA-related CMPs were noted to involve patients arriving to or departing from the ED with law enforcement, and because of variability in detail of summaries we suspect the actual number may be much greater. Among these, the vast majority involved patients with psychiatric emergencies. The CMPs often involved failure to provide an appropriate MSE, stabilization or appropriate transfer. Perspective-taking has been identified as an effective de-biasing technique in other healthcare contexts and may also have broader applicability to improving mutual understanding among hospital staff and LEOs.^18^ Knowledge about aspects of EMTALA requirements has been previously identified as a barrier to EMTALA compliance.^19^ A more nuanced understanding of EMTALA duties among hospital staff can help to ensure that EMTALA obligations are met, ensuring access to emergency care for LEO-involved patients, while maintaining safety for patients, visitors, and staff within the department. Based on themes identified from review of CMPs resulting from EMTALA citations, essential concepts for administrators and ED staff to know are highlighted below and ensure a broader appreciation of the factors that may influence law enforcement actions in EDs.

Patients presenting to the ED accompanied by law enforcement were frequently redirected to alternate facilities without an MSE to evaluate for an EMC, and they were often not entered into the central log. One CMS regulatory requirement related to EMTALA is that patients presenting to a dedicated ED must be entered into a central log (42 CFR 489.20(r)(3)) to provide accountability for fulfilling the EMTALA screening requirement.^20^ The duty to perform an MSE applies to all individuals who request an evaluation for a medical condition, individuals who have an evaluation requested on their behalf by EMS, LEO or another person, or individuals who a prudent layperson would believe require evaluation for an EMC based on the individual’s appearance or behavior. While EMTALA is a duty of the hospital, instances where LEOs were turned away highlight the potential utility of educating LEOs and paramedics about a hospital’s EMTALA duties to facilitate advocacy for patients in scenarios where hospital staff fail to fulfill the hospital duty and attempt to re-route patients without a MSE to alternate facilities.

To ensure compliance with requirements, patients presenting to a dedicated ED must be entered into the central log and provided an appropriate MSE, regardless of whether they are LEO-involved, they will ultimately be criminally charged, or will receive further care at another facility such as a jail. If an appropriate MSE determines that the patient does not have an EMC, or if an identified EMC is ultimately stabilized, the EMTALA obligations end. Similarly, EMTALA obligations end if a patient either does not consent or withdraws consent to be evaluated and treated. Alternatively, if stabilizing the EMC requires capabilities not available at the hospital, the facility must arrange an appropriate transfer to another medical facility.

The most recent iteration of CMS EMTALA interpretive guidelines provides guidance regarding the use of EDs for non-emergency services, including, in some instances, by LEO-involved patients.^21^ For example, if an individual presents to a dedicated ED and requests services that are not for a medical condition, such as preventative services or the gathering of evidence for criminal law cases (eg, sexual assault evidence collection, forensic blood alcohol test), the hospital may not necessarily be obligated to provide an MSE, in the absence of a request for examination or treatment for a medical condition (eg, intoxication, withdrawal, traumatic injury, injuries or exposures resulting from a sexual assault), or if a prudent layperson observer would not believe that the individual needed such examination or treatment.^21^ However, guidelines indicate that if preceding circumstances suggest (eg, a motor vehicle accident or other situation where an individual may have sustained an injury, assault that may require pregnancy or HIV prophylaxis, substance ingestion), or if a prudent layperson observer would believe, that the individual needed such examination or treatment, an MSE to evaluate for the presence of an EMC would be warranted.^21^ Furthermore, interpretive guidelines state that surveyors will evaluate each case on its own merit when determining a hospital’s EMTALA obligation when LEOs request evidence collection for use as evidence in criminal proceedings.^21^

Several LEO-involved patients were noted to have psychiatric emergencies for which clinical staff initiated involuntary holds, which are a function of state law, not federal law. Criteria and process vary considerably state to state, with multiple states requiring LEO and/or judicial involvement.^22^ In such cases, it is important that hospitals and law enforcement collaboratively develop local procedures to meet both the requirements of state involuntary commitment laws and federal EMTALA rules. Our study findings suggest that hospitals may be cited under EMTALA if they fail to use available resources, including on-call mental health specialists. Further guidance on these matters in future iterations of EMTALA interpretive guidelines would be helpful to hospitals and clinicians.

The EMTALA requirements of an appropriate transfer are important to consider as well. EMTALA requires that the transfer of a patient with an EMC be done through qualified personnel and equipment, among other requirements. While transfer to another facility via non-EMS mechanisms, either generally via laypersons private vehicle or via law enforcement, is not explicitly prohibited under EMTALA, hospitals would be wise to consider which qualified personnel and transportation equipment would be required for any given patient and assure evaluation is sufficient to ensure that benefits of such transfer outweigh risks. Stabilizing treatment, within the capabilities of the hospital, must also be provided to mitigate risks of transfer, regardless of ultimate means of transfer. Decisions regarding method of transfer should be individualized, appropriately reasoned, and documented. Authoritative guidance in this area would be helpful as well.

We also identified CMPs in which patients were discharged to jail after they were noted to have behavior described as aggressive and/or violent in the ED. While it is vital for the ED to ensure a safe environment for staff and other patients, and while law enforcement involvement may be necessary for safety and a discharge to law enforcement custody may be the ultimate disposition, the EMTALA obligations continue to apply. All patients must still receive an appropriate MSE and receive stabilizing treatment for identified EMCs. Hospitals would be prudent to ensure the EMTALA obligations are fulfilled prior to any patient being discharged to the custody of law enforcement.

The CMP summaries describe several patients who were escorted from the premises by LEOs after behavior perceived to be aggressive or uncooperative without an adequate MSE/stabilization, and were later found to have primarily medical conditions that may have accounted for their behavior. These serve as a reminder that behavioral issues such as aggression or non-cooperation may result from primary physical health conditions and may also represent an EMC in their own right. Adequate assessment is needed to exclude presence of a medical cause for uncooperative or aggressive behavior. The CMP summaries describing discharge of patients later determined to have unstabilized EMCs to jail due to hospital policies involving alcohol intoxication, for example, also serve as a reminder that the MSE and stabilization requirements of EMTALA are enforceable regardless of any hospital, local, state or regional policies regarding dispositions. It is essential for all ED staff to consider EMTALA requirements when treating patients who present to an ED, regardless of whether the patient is arriving to or departing from the facility with law enforcement.

Many CMPs reviewed highlight the challenge of meeting a hospital’s obligations under EMTALA, along with ensuring the safety of staff, other patients, and visitors under circumstances in which patients may be physically aggressive for a myriad of reasons, potentially due to underlying psychiatric emergencies, or with medical conditions impacting behavior. Further clarification from CMS in future iterations of the EMTALA Interpretive Guidelines may be helpful in guiding hospitals to develop policies and procedures to both ensure compliance with EMTALA, while simultaneously maintaining the safety of patients, visitors, and staff.

## LIMITATIONS

Relatively few EMTALA violations result in a CMP, and our findings are limited to these violations, although this number may not account for CMPs that encompass multiple EMTALA violation events.^10^,^13^ Settlement summaries varied considerably in length and detail across the study period. Therefore, it is possible that LEO-involvement may not have been identified for CMPs where details regarding arrival or departure or patient characteristics were not included (eg, the OIG alleged that the hospital failed to provide appropriate MSEs and stabilizing treatment to two patients). Therefore, described cases represent examples of CMPs with LEO-involvement, but the current study likely underestimates the proportion of LEO-involved clinical cases resulting in EMTALA-related CMPs. Additionally, available CMPs do not always include the full set of EMTALA deficiencies identified, and deficiencies are only called out when negligent violations are noted, perhaps explaining why there were far fewer noted failures to stabilize than failures to complete an MSE.

The CMPs, as reported by OIG, provide an analyzable, albeit limited, narrative summary that enables descriptions of events leading up to an EMTALA violation and the resulting CMP. However, data available for analysis is limited to the unstructured CMP summary of settlement text, which typically contains only brief narratives of the patient’s ED encounter, often lacking the clinical detail and nuance that a clinician-reader might desire. The CMP summaries released by the OIG do not fully capture the context in which EMTALA violations occur, and whereas CMS has made limited redacted inspection text related to some EMTALA events publicly available, plans of corrective action and quality improvement determinations are not routinely publicly released. Attempts to obtain additional information about enforcement responses via the Freedom of Information Act have yielded variable responses. We also want to note that the purpose of this study was to describe how EMTALA has been enforced and not to establish whether CMS or OIG determinations were merited.

## CONCLUSION

Every CMP describing a LEO-involved patient involved a failure to provide an adequate MSE, and two thirds additionally involved failure to provide stabilizing treatment. Findings indicate a need for education, both among hospital staff and LEOs, surrounding EMTALA requirements, regardless of whether the patient is accompanied by or in the custody of law enforcement. Transfers via law enforcement vehicles are not explicitly prohibited but should be carefully evaluated on a case-by-case basis. Findings may serve to guide future, more comprehensive evaluations of the universe of EMTALA citations related to the care of LEO-involved patients and to inform future iterations of interpretive guidelines that may benefit from additional clarification and examples regarding the duties pertaining to LEO-involved individuals for whom care is sought in EDs.

## Supplementary Information



## Figures and Tables

**Table 1:** Characteristics of EMTALA-related civil monetary penalties involving patients arriving to or departing from the emergency department with law enforcement personnel 2002–2023 (N=260).

	Overall		Arriving with LEO		Not arriving with LEO	
Overall Characteristics	N	%	n	%	n	%
CMPs involving LEOs	15	100%	9	60%	6	40%
CMPs with multiple patients and LEO involvement	2	13.3%	0	0%	2	33.3%
Patient Characteristics						
Psychiatric condition	12	80%	8	88.9%	4	66.7%
Medical condition*	4	26.7%	0	0%	4	66.7%
Nature of condition not stated/unclear**	1	6.7%	1	11.1%	1	16.7%
Dispositions and Outcomes						
Discharged without mention of LEO involvement in disposition	3	20%	3	33.3%	0	0%
Discharged to jail with LEO***	4	26.7%	0	0%	4	66.7%
Transported to alternate ED by LEO at direction of ED staff	5	55.6%	5	55.6%	0	0%
Noted to have been admitted at alternate facility	2	13.3%	2	22.2%	0	0%
Disposition from alternate ED not specified	3	20%	3	33.3%	0	0%
Escorted from ED or campus by LEO****	2	13.3%	0	0%	2	33.3%
Disposition unclear	1	6.7%	1	11.1%	0	0%
EMTALA Deficiency Referenced in Penalty Summary						
Failure to provide appropriate MSE	15	100%	9	100%	6	100%
Failure to stabilize	10	67%	5	55.6%	5	83.3%
Failure to arrange appropriate transfer	2	13.3%	2	22.2%	0	0%

* Includes a patient who presented to the ED for a primary psychiatric complaint, who was reportedly injured without apparent medical treatment. Additionally includes a CMP involving patients with suicidality and overdose, and two patients escorted out, due to concerns about behavior. who were ultimately found to have serious medical conditions.

** Includes a CMP with 2 patients sent to jail, 1 without stabilization of a psychiatric emergency, and another without appropriate MSE.

*** One patient who was discharged to jail returned and was admitted for an overdose.

**** EMS transported a patient from a parking lot to another facility where the patient was hospitalized for meningitis.

*CMP*, civil monetary penalty; *LEO*, law enforcement officer; *ED*, emergency department; *EMS*, emergency medical services; *MSE*, medical screening exam.

**Table 2:** Illustrative case 1.

A patient with a history of schizophrenia and a recent hospitalization at a mental health facility presented to an emergency department (ED) with psychosis and homicidal ideation toward his father. The patient was placed on an involuntary psychiatric hold after an emergency physician (EP) determined the patient to be a danger to himself or others. The patient was treated with an antipsychotic and transferred via emergency medical services for higher level of care to the ED of the cited facility for mental health evaluation. The patient was noted to be “combative, belligerent, uncooperative, rambling, delusional, hostile, impulsive, and inappropriate” upon arrival to the accepting ED. The patient became agitated and, despite de-escalation attempts by multiple personnel, struck an off-duty police officer contracted by the hospital for security. The patient sustained injuries as he was being restrained by security staff and handcuffed by the off-duty police officer. The patient was noted to be a threat to the staff. Shortly thereafter, an additional dose of antipsychotic medication was ordered, and the patient was medically “cleared for jail” by the EP with a discharge diagnosis of schizophrenia with acute agitation and a differential diagnosis of polysubstance abuse. The patient was arrested for battery and transported to jail by law enforcement. Following a report of concern for an Emergency Medical Treatment and Labor Act (EMTALA) violation, the Centers for Medicare & Medical Services regional office authorized an investigation by the state. Findings related to that inspection are included below. Within two weeks, the hospital received citations related to EMTALA deficiency tags 2400 (compliance with responsibilities of Medicare-participating hospitals in emergency cases), 2402 (sign posting), 2406 (MSE), and 2407 (stabilizing treatment). The inspection text indicated that the patient presented with manifestations of acute symptoms of sufficient severity such that his condition could reasonably be expected to place the health of the patient in serious jeopardy in the absence of immediate medical attention. Inspection text revealed that there was no documentation to indicate that a mental health evaluation was provided for the patient, and that the hospital failed to ensure that hospital policy was followed as evidenced by failing to ensure that the patient who was involuntarily committed received an appropriate mental health screening exam from the appropriate personnel (on-call psychiatrist) to determine whether or not a psychiatric emergency medical condition existed. Review of hospital records revealed that a psychiatrist was on call and that the facility’s adult psychiatric unit was not at full capacity on the day of the patient’s ED visit. Investigation text revealed that actions taken by the off-duty police officer contracted for security were noted by the contracted security’s account manager to be “more in line with what he/she would have been trained to do as a police officer.” In an interview conducted as part of the inspection, the account manager for the contracted security stated that police officers “use their judgment as police officers,” and that the account manager “could not interfere with them doing their job—they are in their uniform and representing their agency.” The EP recalled that the patient did not receive a mental health evaluation because the physician had been told by security that the patient’s violence at that point was beyond what the facility could safely control. Interview with the ED medical director revealed that an associated critical access hospital sent behavioral health patients to this facility for evaluations because the patients were difficult to manage, and the facility reportedly “lacked security there.” He further stated that in general if a behavioral health patient arrived “swinging/punching, the EP would do a medical screening exam to determine medical clearance. If the patient already had laboratory studies performed and was uncontrollable, they may go to jail, depending on the severity of the signs/symptoms. The police officer was there to protect the staff.” The report indicated that the facility failed to ensure that their policy, “Care of the Patient with Assaultive Behavior,” was followed as evidenced by failing to call a “Code Gray” for the involved patient. The facility also failed to follow their policy related to “Care of the Mental Health Patient” as evidenced by failing to ensure that the mental health screener was called to work collaboratively with patient/family, clinical staff, the EP, and the on-call psychiatrist to appropriately screen the patient and determine appropriate treatment. Two years after the EMTALA citation was issued, the hospital agreed to a $50,000 civil monetary penalty settlement with the OIG to resolve allegations that the hospital had violated EMTALA in failing to evaluate and treat a mentally ill patient transferred from another hospital for involuntary inpatient psychiatric care despite having an on-call psychiatrist and capabilities to treat the patient.

**Table 3:** Illustrative case 2.

Emergency medical services (EMS) responded to a county jail for a 59-year-old male with a chief complaint of seizures. EMS found the patient to be alert and oriented with normal vital signs and transported the patient to an emergency department (ED). Upon arrival, an ED Registered Nurse (RN) informed EMS that the patient had a restraining order at the facility due to previous encounters in which he was combative and aggressive towards staff. EMS stated that they were not aware of the restraining order and that the hospital was the patient’s choice. Per reports, the ED RN instructed EMS to leave the ED with the patient without an medical screening exam (MSE). EMS transported the patient to an alternate ED where he received an MSE and stabilizing treatment, including phenytoin and phenobarbital levels, along with routine labs. The patient was discharged from the ED with instructions to call his physician the following day to manage his medications. Following a report of concern for an Emergency Medical Treatment and Labor Act (EMTALA) violation, the Centers for Medicare & Medical Services regional office authorized an investigation within weeks. Inspection findings are summarized below. Approximately a month after the incident, the hospital received citations related to EMTALA deficiency tags 2400 (compliance with responsibilities of Medicare participating hospitals in emergency cases), 2405 (emergency room log), and 2406 (MSE). Review of facility policy indicated a requirement that a central log be maintained for each individual who “comes to the ED” seeking assistance that documents whether he or she refused treatment, was refused treatment or whether he or she was transferred, admitted and treated, stabilized and transferred or discharged. The facility failed to ensure that their policy and procedure regarding the ED central log was followed when this patient presented to the ED requesting medical assistance. A review of the hospital’s ED central log dated on the day of the incident showed that the patient had never been entered into the Central Log. An interview with the RN involved in the patient’s care stated that the RN recognized the patient and discussed with other ED staff. The RN reported having “assumed that the ‘restraining order’ and the ‘no trespass order’ were the same type of order that meant the patient couldn’t be in the hospital unless in acute distress like postictal, coding, or active seizures,” and reported not being aware that EMTALA applied if a patients had a restraining order. Nearly two and a half years after the original incident, the facility entered into a $40,000 settlement agreement with the Office of the Inspector General to resolve allegations related to this EMTALA violation.

## References

[1] Harada MY, Lara-Millán A, Chalwell LE (2021). Policed Patients: How the Presence of Law Enforcement in the Emergency Department Impacts Medical Care. Ann Emerg Med.

[2] Ding ML, Gerberi DJ, McCoy RG (2023). Engaging emergency medical services to improve postacute management of behavioural health emergency calls: a protocol of a scoping literature review. BMJ Open.

[3] Rosen DL, Travers D (2021). Emergency department visits among patients transported by law enforcement officers. PLoS One.

[4] Wandling MW, Nathens AB, Shapiro MB (2016). Police transport versus ground EMS: a trauma system-level evaluation of prehospital care policies and their effect on clinical outcomes. J Trauma Acute Care Surg.

[5] Khatri UG, Kaufman EJ, Seeburger EF (2023). Emergency physician observations and attitudes on law enforcement activities in the emergency department. West J Emerg Med.

[6] Weiss M, Weiner J (2023). To protect and serve: clarifying the role of law enforcement in the emergency department.

[7] 42 U.S. Code § 1395dd- Examination and Treatment for Emergency Medical Conditions and Women in Labor.

[8] Terp S, Seabury SA, Arora S (2017). Enforcement of the Emergency Medical Treatment and Labor Act, 2005 to 2014. Ann Emerg Med.

[9] Office of Inspector General (OIG) US Department of Health and Human Services (2001). The Emergency Medical Treatment and Labor Act: The Enforcement Process.

[10] Zuabi N, Weiss LD, Langdorf MI (2016). Emergency Medical Treatment and Labor Act (EMTALA) 2002–15: review of Office of Inspector General patient dumping settlements. West J Emerg Med.

[11] McKenna RM, Purtle J, Nelson KL, Regenstein M, Ortega AN (2018). Examining EMTALA in the era of the patient protection and Affordable Care Act. AIMS Public Health.

[12] Terp S, Wang B, Raffetto B (2017). Individual physician penalties resulting from violation of Emergency Medical Treatment and Labor Act: a review of Office of the Inspector General patient dumping settlements, 2002–2015. Acad Emerg Med.

[13] Terp S, Ahmed S, Reichert Z (2024). Civil monetary penalties for EMTALA violations involving minors, 2002–2023. Hosp Pediatr.

[14] Terp S, Wang B, Burner E (2019). Civil Monetary Penalties Resulting From Violations of the Emergency Medical Treatment and Labor Act (EMTALA) Involving Psychiatric Emergencies, 2002 to 2018. Acad Emerg Med.

[15] Terp S, Wang B, Burner E (2020). Penalties for Emergency Medical Treatment and Labor Act Violations involving obstetrical emergencies. West J Emerg Med.

[16] Office of the Inspector General Patient Dumping Websites.

[17] Office of Inspector General (2024). Semiannual Report Archives.

[18] Vela MB, Erondu AI, Smith NA (2022). Eliminating explicit and implicit biases in health care: evidence and research needs. Annu Rev Public Health.

[19] Hsuan C, Horwitz JR, Ponce NA, Hsia RY, Needleman J (2018). Complying with the Emergency Medical Treatment and Labor Act (EMTALA): challenges and solutions. J Healthc Risk Manag.

[20] US Government Publishing Office (2024). Conditions of Participation: Requirements for States and Hospitals 2024.

[21] CMS (2019). Appendix V Interpretive Guidelines- Responsibilities of Medicare Participating Hospitals in Emergency Cases.

[22] Hedman LC, Petrila J, Fisher WH (2016). State laws on emergency holds for mental health stabilization. Psychiatr Serv.

