# Supplementary material for: Civil Monetary Penalties from Violations of the Emergency Medical Treatment and Labor Act for Patients Arriving or Leaving with Law Enforcement
Source: West J Emerg Med. 2025 May 19;26(3):712–9. doi: 10.5811/westjem.39677 (PMC12208041; doi:10.5811/westjem.39677)
Supplement: Supplementary file 1 [file wjem-26-712-s001.docx]

**Supplement A.** Enforcement process diagram.

**
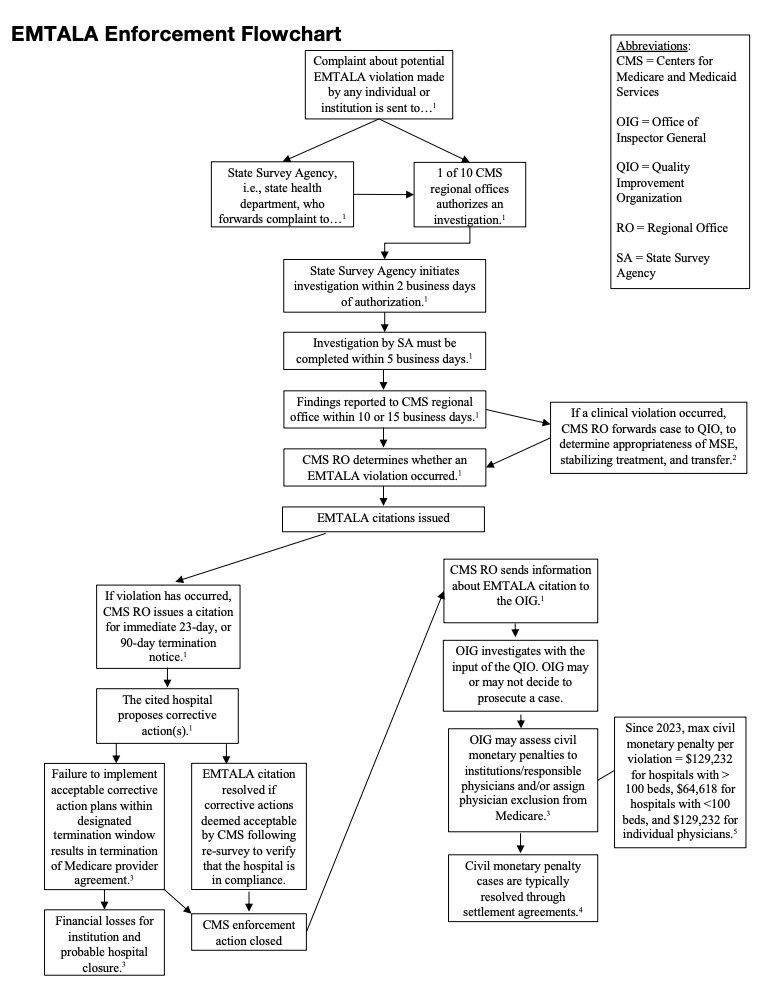
**

**Supplement A References**

1. Centers for Medicare & Medicaid Services. State Operations Manual, Appendix V - Interpretive Guidelines - Responsibilities of Medicare Participating Hospitals in Emergency Cases. 2019, July 19; Retrieved from <https://www.cms.gov/regulations-and-guidance/guidance/manuals/downloads/som107ap_v_emerg.pdf>
2. Centers for Medicare & Medicaid Services. Quality Improvement Organization (QIO) Manual: Chapter 9 - Appeals and Grievances (Rev. 3, 43-78). 2016, February 12; Retrieved from <https://www.cms.gov/Regulations-and-Guidance/Guidance/Manuals/downloads/qio110c09.pdf>
3. Terp S, Seabury SA, Arora S, Eads A, Lam CN, Menchine M. Enforcement of the Emergency Medical Treatment and Labor Act, 2005 to 2014. *Ann Emerg Med*. 2017 Feb;69(2):155-162.e1. doi: 10.1016/j.annemergmed.2016.05.021. Epub 2016 Aug 2. PMID: 27496388; PMCID: PMC7176068.
4. Terp S, Wang B, Burner E, Connor D, Seabury SA, Menchine M. Civil monetary penalties resulting from violations of the Emergency Medical Treatment and Labor Act (EMTALA) involving psychiatric emergencies, 2002 to 2018. *Acad Emerg Med*. 2019 May;26(5):470-478. doi: 10.1111/acem.13710. Epub 2019 Apr 17. PMID: 30994255; PMCID: PMC7237063.
5. Becerra, X. Annual civil monetary penalties inflation adjustment. *Federal Register*. 2023, October 6; Retrieved from <https://www.federalregister.gov/documents/2023/10/06/2023-22264/annual-civil-monetary-penalties-inflation-adjustment>

**Supplement B:** Appendix of EMTALA-related civil monetary penalties noted to involve patients arriving or departing from emergency departments with law enforcement

| 74  A | ***Redacted*,** Missouri, agreed to pay $22,500 to resolve its liability for CMPs under the patient dumping statute. The OIG alleged that ***Redacted*** failed to provide an appropriate medical screening examination or an appropriate transfer to a male that was experiencing a severe psychotic episode who was presented involuntarily by a deputy sheriff to ***Redacted***’s emergency department. The deputy requested assistance for the patient and an involuntary 96-hour hold. An ED nurse, after allegedly consulting with supervisors, informed the deputy that the hospital did not take involuntary holds and offered no further assistance. For the next two hours, the patient waited in the ED while the deputy made arrangements to take him to another hospital 90 miles away where the patient was admitted and treated. |
| --- | --- |
| B | ***Redacted***, California, agreed to pay $40,000 to resolve its liability for CMPs under the patient dumping statute. The OIG alleged that on five separate occasions, the hospital failed to provide an appropriate medical screening examination to five patients. Four of the alleged violations involved patients who left the hospital's emergency department (ED) after waiting three or more hours and without being seen. The fifth alleged violation involved a 37-year-old male who presented to the hospital's ED with a chief complaint of suicidal ideation. The hospital allegedly refused requests from the patient and police to admit the patient and refused police requests to arrange a transfer of the patient to another facility. Ultimately, the police transported the patient to another hospital. |
| C | ***Redacted***, Louisiana, agreed to pay $15,000 to resolve its liability for CMPs under the patient dumping statute. The OIG alleged that ***Redacted*** failed to provide an appropriate medical screening examination to a patient who was suffering from an acute psychotic episode. The patient was brought to ***Redacted*** after being apprehended by local police officers. |
| D | ***Redacted***, Florida, agreed to pay $20,000 to resolve its liability for CMPs under the patient dumping statute. The OIG alleged that ***Redacted*** failed to provide appropriate medical screening examinations and/or stabilizing treatment for two patients who went to ***Redacted***’s emergency department (ED). The mother was allegedly informed that ***Redacted*** did not treat pediatric patients and that she would have to take her daughter to another facility. The daughter, who was pregnant, presented to another facility with lower abdominal pain and vaginal bleeding. She was stabilized and transported to another hospital. The second patient presented to ***Redacted***'s ED accompanied by fire rescue workers, the police, and his grandmother. The patient was threatening to burn himself. A psychiatric nurse allegedly suggested that the patient be taken to another hospital across the street, where beds were readily available, in order to avoid a long wait in the ED The patient was taken to the other hospital where he was admitted. |
| E | ***Redacted***, Missouri, agreed to pay $35,000 to resolve allegations that it failed to provide a medical screening examination and stabilizing treatment for a patient who was brought to ***Redacted***'s psychiatric unit by his parents. The patient was a young adult with a history of schizophrenia. His psychiatrist had made arrangements for the patient to be treated and hospitalized at ***Redacted***. While waiting to be screened, he was placed unaccompanied in an assessment room. Later he walked out of the room and struck a male staff member. He was then placed in a seclusion room and, without any assessment or treatment, was taken to jail where he was held overnight. |
| F | ***Redacted***, Vermont, agreed to pay $50,000 resolve its liability for Civil Monetary Penalties under the patient dumping statute. OIG alleged that: (1) ***Redacted*** failed to provide stabilizing treatment to a patient with an emergency psychiatric condition before having him criminally charged and transferred to jail; and (2) ***Redacted*** failed to provide an appropriate medical screening examination to a second patient before having him criminally charged and sent to jail. |
| G | ***Redacted***in South Carolina entered into a settlement agreement with the Office of Inspector General (OIG) for the U.S. Department of Health and Human Services to resolve claims that it failed to provide stabilizing treatment to a patient in one of its emergency rooms. Specifically, OIG alleged that on ***Redacted***, a 58-year-old male patient, who was incarcerated at the time, was transported by an Emergency Medical Services (EMS) ambulance to ***Redacted***, a ***Redacted*** facility. EMS contacted emergency room personnel to inform them of the patient's transport but, when the patient arrived at the emergency room, a nurse informed EMS personnel that the medical center could not treat the patient because ***Redacted*** had a "no trespass" order on him. EMS then took the patient to a nearby hospital, and ***Redacted*** never provided a medical screening examination of the patient. This $40,000 settlement resolves ***Redacted*** 's civil monetary penalties liability under the patient dumping statute. |
| H | ***Redacted***, in Florida, entered into a $25,000 settlement agreement with OIG. The settlement agreement resolves allegations that ***Redacted*** violated the Emergency Medical Treatment and Labor Act (EMTALA) when it failed to provide an appropriate medical screening examination and stabilizing treatment for a 42-year-old female. OIG's investigation revealed the following. The patient arrived at ***Redacted*** 's emergency department complaining of headaches, right arm pain and diarrhea the previous day. She was seen by a physician's assistant and then asked to wait in the waiting room. While there, she vomited and continued to complain of right arm pain. The physician's assistant concluded that she did not need immediate medical attention and asked the patient to leave the emergency department. When the patient resisted and her family complained, the emergency department personnel called the police to escort her out of the emergency department. After unsuccessful attempts by police and paramedics to get the patient into a car, her family requested the emergency department call an ambulance so she could be taken to another hospital. When the ambulance arrived, the patient was unresponsive and taken to another hospital where she was placed on a ventilator in the Intensive Care Unit and later diagnosed with bacterial meningitis. Under EMTALA, a small hospital can be fined up to $25,000 per violation. Senior Counsel ***Redacted*** represented OIG. |
| I | ***Redacted*** in Georgia Settles Case Involving a Patient Dumping Allegation. On ***Redacted***, ***Redacted***, in Georgia, entered into a $50,000 settlement agreement with OIG. The settlement agreement resolves allegations that ***Redacted*** violated the Emergency Medical Treatment and Labor Act (EMTALA) when it failed to evaluate and treat a mentally ill patient who was transferred from another hospital to ***Redacted*** for involuntary inpatient psychiatric care. OIG's investigation revealed the following. The patient was aggressive and combative upon his arrival to ***Redacted***'s emergency department. Three security personnel, including an off-duty police officer working for ***Redacted***, attempted to restrain the patient while a nurse went to retrieve medication to calm him down. When the security personnel entered the room, the patient attempted to strike one of them. In response, a security officer hit the patient in the head and pushed him until he fell on the bed. The security officers then wrestled the patient to the ground and handcuffed him, causing injury to the patient. When the nurse returned, the security personnel informed her that the patient's behavior was beyond what ***Redacted*** could safely control. Without psychiatric evaluation or appropriate medical treatment, the emergency department physician medically cleared the patient, and he was taken to jail. Despite having an on-call psychiatrist and capabilities to treat the patient, at no point was he evaluated or treated by a mental health professional. Under EMTALA, hospitals can be fined up to $50,000 per violation. Senior Counsel ***Redacted*** represented OIG. |
| J | ***Redacted*** in Georgia Settles Case Involving a Patient Dumping Allegation. On ***Redacted***, ***Redacted***, in Georgia, entered into a $40,000 settlement agreement with OIG. The settlement agreement resolves allegations that ***Redacted*** violated the Emergency Medical Treatment and Labor Act (EMTALA) when it failed to provide an adequate medical screening examination and stabilizing treatment to a patient. OIG's investigation revealed the following. The patient was extracted from his apartment by a SWAT team and brought to ***Redacted*** 's emergency department (ED) by a police officer due to complaints of suicidal and homicidal ideations. While at ***Redacted***, two Licensed Professional Counselors (LPCs) evaluated the patient and determined that the patient should be held involuntarily for further evaluation and treatment. Approximately five hours after the patient's arrival in the ED, the ED physician discharged the patient without consulting the LPCs or the on-call psychiatrist. Under EMTALA, hospitals can be fined up to $50,000 per violation. Senior Counsel ***Redacted*** and Associate Counsel ***Redacted*** represented OIG. |
| K | ***Redacted***, in South Carolina, entered into a $1,295,000 settlement agreement with OIG. The settlement agreement resolves allegations that, in 36 incidents investigated by OIG, ***Redacted*** violated the Emergency Medical Treatment and Labor Act (EMTALA). In these incidents, individuals presented to ***Redacted***'s Emergency Department (ED) with unstable psychiatric emergency medical conditions. Instead of being examined and treated by an on-call psychiatrist, and despite empty beds in its psychiatric unit to which the patients could have been admitted for stabilizing treatment, the patients were involuntarily committed and kept ***Redacted***'s ED for between 6 and 38 days each. The following is an example of one such incident. A patient presented to ***Redacted***‘s ED via law enforcement with psychosis and homicidal ideation and was involuntarily committed. The patient did not receive psychiatric examination or treatment by available ***Redacted*** psychiatrists and was not admitted to the psychiatric unit for stabilizing treatment. Instead, the patient was kept in the ED for 38 days and at one point was seen by a psychiatrist from another facility that was familiar with her condition. The psychiatrist prescribed a variety of medications for agitation. The patient eventually was discharged home. Senior Counsel ***Redacted*** represented OIG. |
| L | ***Redacted***, Massachusetts, entered into a $60,000 settlement agreement with OIG. The settlement agreement resolves allegation that ***Redacted*** violated the Emergency Medical Treatment and Labor Act when it failed to provide an appropriate medical screening examination for a fourteen-year-old patient and inappropriately transferred her to another hospital. The patient arrived at ***Redacted***’s emergency department by ambulance, secured to a stretcher and under police escort, for psychiatric evaluation after combative behavior at home and banging her head against a wall. Upon arrival at ***Redacted*** the patient was placed in a room, still secured to the stretcher. ***Redacted***’s emergency department physician came into the room and told the paramedics that the patient should be transported to ***Redacted***’s emergency department for pediatric psychiatric evaluation. Before recommending transfer, ***Redacted*** failed to provide the patient with a medical screening exam. On route to ***Redacted***, the police instructed the ambulance to take the patient to a different hospital where her mother was waiting. Senior Counsel ***Redacted*** represented OIG. |
| M | ***Redacted***, Missouri, entered into a $100,000 settlement agreement with OIG. The settlement agreement resolves allegations that ***Redacted*** violated the Emergency Medical Treatment and Labor Act when it failed to provide an adequate medical screening examination and stabilizing treatment for two patients who presented to ***Redacted***’s Emergency Department (ED) in 2011. OIG alleged that instead of being properly evaluated and treated, the patients were discharged with unstabilized emergency medical conditions to the custody of police pursuant to a hospital policy: if a patient had a blood alcohol level (BAL) above 100, the patient was given to local law enforcement and taken to jail. The first patient was 25 years old when she called a crisis hotline and an ambulance was dispatched to her residence. She was transported to ***Redacted***'s ED for evaluation of a possible suicide attempt by overdose. The patient's BAL was 422 and the ED physician discharged her into the custody of local law enforcement where she was detained in jail and expected to see a counselor. The second patient was 41 years old when he presented to ***Redacted*** after attempting suicide by overdose. The patient was depressed, had a history of psychiatric problems, and had recently been admitted for electroconvulsive therapy. The patient's BAL was 288 and he was discharged into the custody of local law enforcement and taken to jail. The next day the patient was seen by a counselor in jail and then released from custody. The patient returned to ***Redacted*** that evening after again attempting suicide by overdose. The patient had slurred speech, was lethargic and had a flat affect and was admitted to the intensive care unit in guarded condition. Senior Counsel ***Redacted*** represented OIG. |
| N | ***Redacted*** Medical Center - ***Redacted***, Nebraska, entered into a $60,000 settlement agreement with OIG. The settlement agreement resolves allegations that, based on OIG’s investigation, ***Redacted*** violated the Emergency Medical Treatment and Labor Act (EMTALA) when it failed to provide an appropriate medical screening examination and stabilizing treatment to a patient. On September 10, 2017, the patient, a 32-year-old male with a history of anxiety and bipolar disorder, presented by ambulance to ***Redacted***’s Emergency Department (ED) at 11:27 a.m. with altered mental status. The patient was accompanied by law enforcement who found him wandering on a nearby college campus where he was knocking property over, attempting to light property on fire, and was walking around with a steering wheel from a car pretending to be driving. The patient’s speech was incoherent, he appeared and smelled poorly kempt and unclean, and he arrived at the ED in wet clothes. ED staff ordered urine and blood toxicology tests for the patient, which returned negative for alcohol and drug intoxication. After yelling and making threatening gestures at ED staff, the patient was handcuffed to his hospital bed. An ED physician ordered Haldol and Ativan for the patient for sedation and instructed that he be discharged when able to ambulate. At 8:02 p.m., the patient was discharged with a diagnosis of altered mental status and mental health disorder and was instructed to follow-up with a mental health professional. At the time of discharge, the patient was still suffering from an emergency medical condition. ***Redacted*** failed to provide the patient with an appropriate medical screening examination and stabilizing treatment within the capabilities of ***Redacted*** prior to discharging him. Senior Counsel ***Redacted*** represented OIG. |
| O | ***Redacted*** Hospital, Illinois, entered into a $85,000 settlement agreement with OIG. The settlement agreement resolves allegations that, based on OIG’s investigation, ***Redacted*** violated the Emergency Medical Treatment and Labor Act (EMTALA) when it failed to provide an appropriate medical screening examination to patient ***Redacted***. On ***Redacted***. Specifically, ***Redacted*** failed to register the patient upon arrival to the triage area in the emergency department (ED) accompanied by paramedics and, when the patient began exhibiting aggressive behaviors, security officers escorted the patient out of the emergency department and prevented the patient’s re-entry. Nursing staff spoke to the patient while the patient was outside the ED but the ED physician was not alerted of the patient’s arrival and no assessment of the patient was performed by ED personnel prior to law enforcement personnel removing the patient from the premises. The patient was brought back to the ***Redacted*** ED approximately five hours later on ***Redacted***, after suffering cardiac arrest at the patient’s home. Efforts to resuscitate the patient were unsuccessful and the patient expired. Deputy Branch ***Redacted*** represented OIG. |

*CMP*, civil monetary penalty; *ED*, emergency department; *EMS*, emergency medical services; *OIG*, Office of the Inspector General.

**Supplement C.** Appendix of case where hospital staff threatened to involve law enforcement if a patient entered the emergency department.

| **Supplementary Exhibit C.** An example of a civil monetary penalty settlement involving the threat of law enforcement involvement. This case was not included in the main analysis. |
| --- |
| CMP Summary |
| ***Redacted,*** a small hospital in South Carolina, entered into a $20,000 settlement agreement with OIG. The settlement agreement resolves allegations that ***Redacted*** violated the Emergency Medical Treatment and Labor Act when it failed to provide an appropriate medical screening examination and stabilizing treatment of a patient who presented to ***Redacted*** after being assaulted and hit in the head. The patient resisted efforts by his mother to get him into a wheelchair to enter the emergency department (ED). Security guards observed the patient's behavior and told the patient's mother that if she brought her son into the ED the guard would have him locked up. The OIG alleges that the mother explained to the security guard that her son had been hit in the head and was bleeding and that a guard allegedly answered by reasserting that he would call the police if her son entered the ED At that point the mother left with her son and later took him to another hospital for evaluation and treatment. Senior Counsel ***Redacted*** represented OIG. |

*ED*, emergency department; *EMS*, emergency medical services; *OIG*, Office of the Inspector General.
